# Uphill treadmill running does not induce histopathological changes in the rat Achilles tendon

**DOI:** 10.1186/1471-2474-14-90

**Published:** 2013-03-11

**Authors:** Rachel C Dirks, Jeffrey S Richard, Angela M Fearon, Alexander Scott, Lauren G Koch, Steven L Britton, Stuart J Warden

**Affiliations:** 1 Center for Translational Musculoskeletal Research, School of Health and Rehabilitation Sciences, Indiana University, Indianapolis, IN, USA; 2 Department of Anatomy and Cell Biology, Indiana University School of Medicine, Indianapolis, IN, USA; 3 Department of Physical Therapy, School of Health and Rehabilitation Sciences, Indiana University, 1140 W. Michigan St., CF-326, Indianapolis, IN, 46202, USA; 4 Centre for Hip Health and Mobility, Vancouver Coastal Health and Research Institute, Vancouver, BC, Canada; 5 Department of Physical Therapy, University of British Columbia, Vancouver, BC, Canada; 6 Department of Anesthesiology, University of Michigan Medical School, Ann Arbor, MI, USA

**Keywords:** Animal model, Tendinitis, Tendinopathy, Tendinosis, Overuse

## Abstract

**Background:**

The purpose of this study was to investigate whether uphill treadmill running in rats created histopathological changes within the Achilles tendon consistent with Achilles tendinosis in humans.

**Methods:**

Twenty-six mature rats selectively bred for high-capacity running were divided into run and cage control groups. Run group rats ran on a treadmill at a 15° incline for a maximum duration of 1 hr/d, 5 d/wk for 9 weeks at increasing speeds, while rats in the cage control group maintained normal cage activity. After 9 weeks, Achilles tendons were harvested for histological processing and semi-quantitative histopathological analysis.

**Results:**

There were no significant group differences within each of the individual histopathological categories assessed (all p ≥ 0.16) or for total histopathological score (p = 0.14).

**Conclusions:**

Uphill treadmill running in rats selectively bred for high-capacity running did not generate Achilles tendon changes consistent with the histopathological presentation of Achilles tendinosis in humans.

## Background

The Achilles tendon connects the muscles of the calf (gastrocnemius and soleus) to the calcaneus, and is the largest and strongest tendon in the body. Functioning to transmit the muscle contractile forces necessary for human stance and locomotion, the Achilles tendon must be able to withstand large-magnitude tensile loads. Although structurally designed to withstand these loads, injuries of the Achilles tendon thought to result from repetitive loading are common [1-5]. The most common of these injuries is termed Achilles tendinopathy (*tendo–* = tendon, *–pathy* = disease).

Achilles tendinopathy refers to a clinical condition characterized by activity-related Achilles tendon pain associated with focal tendon tenderness and intratendinous imaging changes. The underlying pathology has historically been thought to be one of inflammation and the condition has traditionally been labeled as ‘Achilles tendinitis’. However, histopathological studies have consistently shown the pathology underlying tendinopathy to be one of progressive tendon degeneration (tendinosis) rather than inflammation (tendinitis) [6-8]. Thus, the use of the term tendinosis is preferred when describing the pathology associated with Achilles tendinopathy.

Despite consistent identification of the pathology underlying Achilles tendinopathy, little is known about the pathological process/es taking place within the tendon. This limited knowledge has restricted treatment options, with clinical management presently being more of an art than a science. In order to address this void, a suitable animal model of Achilles tendinosis is required. As knowledge regarding human Achilles tendinosis is currently centered around its histopathological features, a suitable animal model is one in which the histopathological features of the injured animal Achilles tendon replicate those observed in the human condition.

Treadmill running represents a potential means of repetitively loading tendons in rats to induce histopathological changes. Although established for the generation of supraspinatus tendinosis [9], treadmill running has had variable success in developing tendinosis-like changes in rat Achilles tendons [10-15]. Soslowsky and colleagues [13] used the same downhill (10º decline) treadmill running program (17 m/min, 1 hr/d, 5 d/wk for up to 16 weeks) as they used to induce supraspinatus tendinosis in an unsuccessful attempt to induce mechanical and geometric changes within the rat Achilles tendon. A possible explanation for the lack of an effect may be that downhill running in quadrupeds results in a forward shift of the center of mass [16] resulting in increased forelimb loading (and elevated subacromial compression) combined with a relative decrease in hindlimb loading.

Glazebrook et al. [11] replicated the same running program as Soslowsky and colleagues [13], but furthered the work by running rats uphill (10º incline) rather than downhill for up to 12 weeks. Uphill running requires the calf muscles (and other antigravity muscles) to contract concentrically to raise the center of mass with each step. The net result may be increased Achilles tendon loading as the increased muscle forces are transmitted to the skeleton. Glazebrook et al. [11] showed uphill running resulted in histological changes consistent with human Achilles tendinosis, including a reduction in collagen organization and an increase in tenocyte number [11]. However, the latter observations were not replicated by Heinemeier et al. [12] who completed a comprehensive study using the same uphill running program, but with the modification increased running speed (20 m/min).

The aim of this study was to build upon these previous studies and investigate whether uphill treadmill running at a higher (15º) incline and speed (up to 30 m/min) creates histopathological changes within the rat Achilles tendon consistent with Achilles tendinosis in humans. Specifically, we assessed the effect of uphill treadmill running on Achilles tendon calcification, adipocytes, synovium attached to the tendon, collagen arrangement, tenocyte morphology, cellularity, and vascularization in rats selectively bred for high-capacity running (HCR).

## Methods

### Ethics statement

All procedures were performed following *a priori* approval from the Indiana University Institutional Animal Care and Use Committee (Animal Welfare Assurance #A4091-01).

### Animals

Twenty-six male HCR rats (age = 24.8 ± 3.2 wk; weight = 374.0 ± 30.3 g) were acquired from the University of Michigan (Ann Arbor, MI) and acclimated for 2 weeks. HCR rats have been artificially selected for aerobic capacity from a founder population of genetically heterogeneous N:NIH rats [17]. Animals in the current study were from the 26th generation of HCR rats, with this strain of rat being used due to their known ability to run long distances. All animals were maintained under standard conditions and provided *ad libitum* access to food and water.

### Treadmill running

Animals were randomly divided into two groups: cage control (n = 11) and running (n = 15). Rats in the cage control group maintained normal cage activity throughout the duration of the study. Rats in the running group ran 5 d/wk for 9 weeks on a treadmill at a 15° incline. Rats were acclimated to the treadmill initially starting with 5 minutes at 10 m/min. The duration and speed of running were gradually increased throughout weeks 1 and 2 until the rats were running for 60 minutes at 25 m/min (Table 1). The duration was kept constant for the remainder of the study while speed was progressively increased up to 30 m/min by the final week of running.

**Table 1:** Running protocol used for the running group of rats

		**Duration (min)**	**Speed (m/min)**
Week 1	Day 1	5	10
	Day 2	5	10
	Day 3	5	10-15
	Day 4	5	10-25
	Day 5	30	15-25
Week 2	Day 6	45	15-25
	Day 7	50	15-25
	Day 8	55	15-25
	Day 9	60	15-25
	Day 10	60	15-25
Week 3		60	15-25
Week 4		60	20-25
Week 5		60	20-25
Week 6		60	20-25
Week 7		60	20-27.5
Week 8		60	20-27.5
Week 9		60	20-30

Hyphenated values in the speed column represent the gradual increase in speed at the beginning of each running session. Running began at the lower speed and was increased by 1 m/min each minute until the higher speed was achieved. Rats ran at this higher speed for the remainder of the duration.

### Histology

Animals were euthanized after 9 weeks of the running regimen, and one of their Achilles tendons was harvested and fixed in 10% neutral buffered formalin for 48 hours before being transferred to 70% ethanol. Tendons were embedded in paraffin, and 6 μm thick midsubstance sagittal sections were cut using a microtome and stained with hematoxylin and eosin. Sections were viewed on a Zeiss Axiophot light microscope and tendon damage assessed using a modified Bonar histopathology scale [18-20]. The entire slide was assessed for the presence of adipocytes, synovial lining, and calcification. These three characteristics were graded either 0 (not present) or 1 (present). Following this assessment, the most pathological region of the tendon was determined based upon collagen arrangement under polarized light. In this region, tendons were graded for collagen arrangement (one field of view at 100x), tenocyte morphology (four fields of view at 200x), cellularity (one field of view at 100x), and vascularity (up to 10 fields of view at 400x). A greater number of fields were viewed for characteristics requiring assessment at higher magnification in an effort to grade a similar total area of tissue for each outcome. The characteristics were graded between 0 (normal) and 3 (maximum pathology). The sum of all 7 categories gave a completely normal tendon a score of 0 and a tendon with maximum damage a score of 15. Samples were randomized and graded by two independent blind examiners (R.C.D. and A.M.F.). Discrepancies in scoring were resolved by discussion, with a third examiner (A.S.) being consulted when consensus could not be reached.

### Statistics

Statistical analyses were performed using the Statistical Package for Social Sciences (SPSS 19.0; IBM) software, with tests being two-tailed with a level of significance set at 0.05. Kolmogrov-Smirnov and Levene tests were used to test for the presence of a normal distribution and homogeneity of variance, respectively. Mann–Whitney U tests were used to compare histopathological scores between groups because the data did not meet the assumptions required for parametric statistics.

## Results

**Figure 1:**
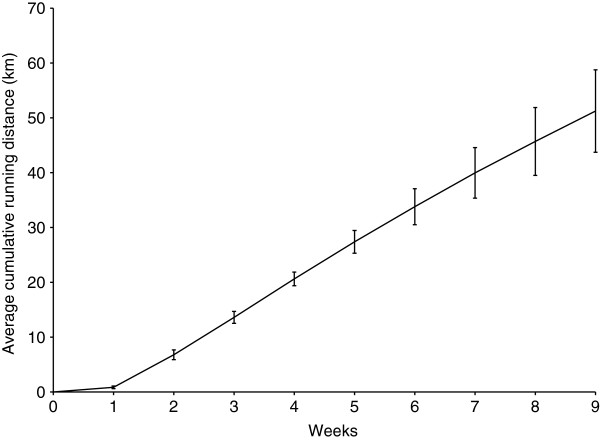
**Cumulative distance ran on the treadmill by rats in the run group.** Error bars represent standard deviation.

**Figure 2:**
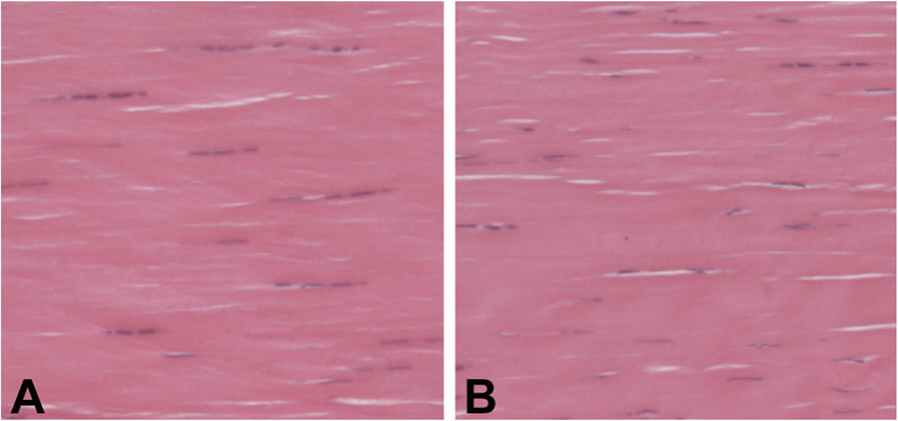
**Representative photomicrographs of the Achilles tendon in the A) run group and B) control group.** Note the uniform appearance of tightly packed, well-aligned collagen fibrils with interspersed, spindle-shaped tenocytes aligned parallel to the fibrils in the tendons from both groups (Stain = Hematoxylin & Eosin; Magnification = 500x).

Animals in the running group ran on the treadmill an average of 51.2 ± 7.5 km during the study (Figure 1). Tendons from the cage control and running groups were similar on gross histological appearance (Figure 2). There was 70-80% agreement in initial grade between examiners for each histological characteristic. There were no group differences within each of the individual histopathological categories assessed (all p > 0.16) or for total histopathological score (p = 0.14) (Table 2).

**Table 2:** Differences (mean ± SD) in individual histopathological categories and total histopathological score in Achilles tendons from cage control and run groups

**Histopathological characteristic**	**Cage control**	**Run**	**p-value**
Adipocytes	0.09 ± 0.30	0 ± 0	0.69
Synovial lining	0.64 ± 0.50	0.57 ± 0.51	0.74
Calcification	0 ± 0	0 ± 0	1.00
Collagen arrangement	1.91 ± 0.83	1.70 ± 0.70	0.38
Tenocyte morphology	0.91 ± 0.70	0.78 ± 0.60	0.66
Cellularity	1.37 ± 0.50	1.00 ± 0.60	0.16
Vascularity	0.91 ± 0.83	0.78 ± 0.95	0.61
Total score	5.82 ± 1.94	4.83 ± 1.83	0.14

## Discussion

Histopathological evaluation of the tendon specimens failed to differentiate between the control rats and the running rats. These data suggest that uphill treadmill running in rodents may not be a suitable animal model for the study of human Achilles tendinosis. Achilles tendinosis in humans is characterized by tissue degeneration with a failed reparative response [6-8]. These changes are identified histologically as collagen fiber disorganization, hypercellularity with atypical tenocyte proliferation and morphology, and neovascularization [7,21,22]. We did not observe changes in these or other individual histopathological categories for human Achilles tendinosis in the current animal study.

Uphill treadmill running in rats has had variable success in producing tendinosis-like changes in the Achilles tendon, with some investigators reporting preliminary tendinosis-like changes [10,11,15] whereas others reporting no evident pathology [12]. The current study used a running program (15° incline with speeds of up to 30 m/min) seemingly more intense than these previous studies, but was unable to find histopathological evidence of Achilles tendinosis. These findings support those of Heinemeier et al. [12] who found that an uphill running program had no effect on the histological appearance and actually improved some mechanical properties of the rat Achilles tendon. These cumulative data suggest that uphill treadmill running in isolation in rats is unable to induce the same histopathological changes as observed in human Achilles tendinosis. Ng et al. [14] recently described a unique bipedal running model wherein rats ran at 17 m/min on a treadmill at a 20° decline, but with the animals in an upright posture. This model resulted in a decrease in Achilles tendon mechanical properties as well as histological changes associated with human Achilles tendinosis; however, the model has yet to be replicated.

One of the strengths of our study was the age of the animals which were older than those used in previous studies. The use of older animals may facilitate the development of tendon pathology if it occurs. Another strength of our study was the use of animals selectively bred for aerobic capacity. The use of HCR rat enabled us to run our animals at a greater incline and at a faster pace than in previous studies, with the intent that these parameters would potentiate the generation of Achilles tendon degeneration. However, this study strength may also be a weakness as the selective breeding of our animals for aerobic capacity may also have led to the development of a tendon phenotype that enhanced tendon resistance to degeneration. Similarly, our study was limited by its relatively small number of animals and limited outcome measures. We do not believe increasing our sample size would have altered the study conclusions as the total histopathological score in treadmill ran rats in the current study were actually about 25% better than in cage controls. Similarly, we do not believe including additional outcome measures are indicated at this stage as histological tendon changes are considered the cardinal sign of the human condition we were attempting to replicate. A final limitation of the current study was the relatively prolonged treadmill acclimation period which lasted 2 weeks and subsequent relatively short period (7 weeks) of running at full speed and duration. These factors may have potentiated tendon adaptation to running and/or limited the ability to produce detectable pathology.

## Conclusions

In summary, we were unable to identify histopathological changes in the Achilles tendon of rats that ran uphill on a treadmill. These cumulative data suggest that uphill running in isolation in rats is unable to induce the same histopathological changes as observed in human Achilles tendinosis.

## Competing interests

The authors declare that they have no competing interests.

## Authors’ contributions

RCD and SJW conceived the project; LGK and SLB provided animals; RCD, JSR and SJW performed treadmill running; RCD, AMF and AS performed histological assessments; RCD wrote the first draft of the manuscript. All authors participated in the trial design, provided feedback on drafts of the manuscript, and read and approved the final manuscript.

## Pre-publication history

The pre-publication history for this paper can be accessed here:

http://www.biomedcentral.com/1471-2474/14/90/prepub
